# Integrating gene expression and GO classification for PCA by preclustering

**DOI:** 10.1186/1471-2105-11-158

**Published:** 2010-03-26

**Authors:** Jorn R De Haan, Ester Piek, Rene C van Schaik, Jacob de Vlieg, Susanne Bauerschmidt, Lutgarde MC Buydens, Ron Wehrens

**Affiliations:** 1 Institute for Molecules and Materials, Analytical Chemistry, Radboud University Nijmegen, Heyendaalseweg 135, 6525 AJ Nijmegen, The Netherlands; 2 Department of Applied Biology, Faculty of Science, Radboud University Nijmegen, Heyendaalseweg 135, 6525 AJ Nijmegen, The Netherlands; 3 MSD, Molenstraat 110, 5340 BH, Oss, The Netherlands; 4 Centre for Molecular and Biomolecular Informatics, Nijmegen Centre for Molecular Life Sciences, Radboud University Nijmegen, Geert Grooteplein 28, 6525 GA, Nijmegen, The Netherlands

## Abstract

**Background:**

Gene expression data can be analyzed by summarizing groups of individual gene expression profiles based on GO annotation information. The mean expression profile per group can then be used to identify interesting GO categories in relation to the experimental settings. However, the expression profiles present in GO classes are often heterogeneous, i.e., there are several different expression profiles within one class. As a result, important experimental findings can be obscured because the summarizing profile does not seem to be of interest. We propose to tackle this problem by finding homogeneous subclasses within GO categories: preclustering.

**Results:**

Two microarray datasets are analyzed. First, a selection of genes from a well-known *Saccharomyces cerevisiae *dataset is used. The GO class "cell wall organization and biogenesis" is shown as a specific example. After preclustering, this term can be associated with different phases in the cell cycle, where it could not be associated with a specific phase previously. Second, a dataset of differentiation of human Mesenchymal Stem Cells (MSC) into osteoblasts is used. For this dataset results are shown in which the GO term "skeletal development" is a specific example of a heterogeneous GO class for which better associations can be made after preclustering. The Intra Cluster Correlation (ICC), a measure of cluster tightness, is applied to identify relevant clusters.

**Conclusions:**

We show that this method leads to an improved interpretability of results in Principal Component Analysis.

## Background

With the advent of large gene expression experiments, new methods of analysis have become necessary to extract relevant information from the data. Exploratory data analysis methods like cluster analysis are regularly used to examine the expression profiles [1-3]. Other methods use annotation information and look for overrepresentation in sets of significantly regulated genes [4-6]. A next step would be to associate relevant profiles with annotation information and experimental variables simultaneously. In this paper we will show advances in finding associations between annotation categories and experimental variables in microarray experiments.

One of the most extensive and systematic methods of categorizing information about genes is the Gene Ontology (GO) database [7]. A problem when relating GO classes with expression profiles is the fact that the genes in these functional classes can have diverse expression profiles. This could mean that a class is not responding to the experimental factors and is not related to the specific biological settings. However, a second possibility is that interesting subgroups are silenced by other heterogeneous or anti-correlated expression profiles present within the class. This may obscure interesting relations. To address this problem, we propose to cluster the expression profiles of genes in every category, and select relevant clusters before applying Principal Component Analysis (PCA; [8]).

PCA has been applied frequently to explore the microarray data in a low-dimensional space [9,10]. Either genes or arrays are described with so called Principal Components, in order to assess relations between arrays or to identify genes with similar expression profiles. The technique is very versatile and can easily cope with large datasets. Work done by Alter et al. [11] is an example of the application of PCA to reduce the dimensionality of microarray data. PCA was applied to the Yeast Cell Cycle dataset of Spellman et al. [12], with each gene as an individual object. We will use the same dataset, but will focus on improvements in the application of PCA to find relations between specified classes of genes and phases in the cell cycle. The work by Goeman et al. [13] is an example of the direct association between annotation information and data analysis. A global test is introduced, determining the relation between a global expression pattern of a group of genes and a clinical outcome of interest. The global expression pattern summarising a group of genes is a method to perform research, based on previous research stored in databases like for instance GO. A second example of summarization of annotation categories is from Chen and Wang [14]. In this paper, gene expression data with prior biological knowledge are integrated by constructing "supergenes" for each gene category by summarizing information from genes related to outcome using a modified principal component analysis (PCA) method. Instead of using genes, these supergenes representing information from each gene category were used in further analysis. Both methods [13,14] indicate that analysing the data on the level improves the results of predictions.

**Figure 1:**
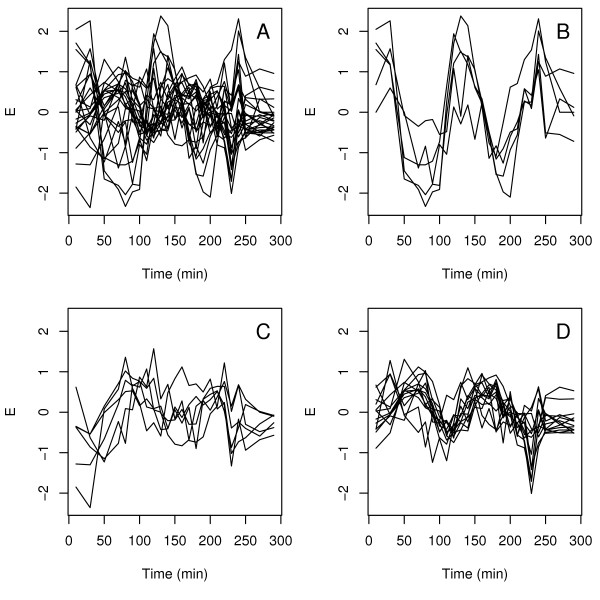
**Example of heterogeneous expression profiles within a single GO class**. Expression profiles for genes annotated with the term GO:0007047 are depicted here for the cdc15 synchronization of the *Saccharomyces cerevisiae *Cell Cycle dataset [12]. From the first subpicture, containing all profiles simultaneously (A), it is clear that there is big variation within the profiles. More homogeneous subgroups, with shifted or even anti-correlated profiles in time, can be identified (B, C, D).

Here, we show that summarizing a GO category in a single profile or supergene can give problems for certain classes, and can be improved. An example of a heterogeneous GO category is shown in Figure 1. The expression data are from the *Saccharomyces cerevisiae *dataset [12] and all the profiles belonging to the genes annotated with GO:0007047 ("Cell wall organization and biogenesis") are shown in Figure 1A. At first glance, there are several particular profiles distinguishable, but it would be hard to give a suitable general description for this category. Previously, Busold et al. [15] also included GO information in their analysis and touched upon this problem by discarding of the categories like the one described here. Thus with the criteria of Busold et al. [15] this category would not be included in the analysis, because the whole category has a low mean correlation over all the genes. The fact that this category is discarded is surprising, because organization of the cell wall is expected to be important in the cell cycle.

We propose to perform clustering of expression profiles present in individual GO classes *before *applying PCA. This is a reversal of the order with respect to more common analyses, where clusters of genes with similar expression profiles are mapped to GO terms. An indication of the improvements of GO class descriptions after cluster analysis is given in Figure 1B, C and 1D. After clustering with model-based clustering [16] these three new subgroups with more distinct profiles can be formed for this class. The new subclasses will not be discarded because the mean correlation of each newly formed class is higher and the role of this process will not be obscured any longer.

The advantages of the cluster analysis will be shown by comparing results from PCA performed with and without preclustering. The *Saccharomyces cerevisiae *dataset and a Mesenchymal Stem Cells (MSC) dataset will be used. The intra-class correlation of clusters will be analyzed to identify better defined subgroups, and two specific examples of GO categories benefiting from the clustering will be given.

## Methods

All calculations are performed in R [17]. GO information is obtained from the R data packages "Yeast" and "hgu133a".

### Datasets

Two datasets are used to show the advantages of the method proposed here. The first dataset is the well known *Saccharomyces cerevisiae *Cell Cycle dataset of Spellman et al. [12], from here on referred to as the Yeast Cell Cycle (YCC) dataset. The focus is on a subset of 800 genes involved in the cell cycle [12]. From this set, genes are selected for which GO annotation information is available. Only GO classes which contain at least 4 genes are considered, which are members of the Biological Process definition in GO. The 24 time points of the cdc15 synchronization method in the experiment are used. As a result of these choices, a data matrix of 24 time points by 348 genes is obtained.

The second gene expression experiment analyzed in this paper, was performed on human mesenchymal stem cells, triggered to undergo osteogenic differentiation (E. Piek et al., manuscript in preparation). This is a time series dataset with multiple osteogenic treatments: Dexamethasone (DEX), Bone Morphogenetic Protein (BMP), Vitamin D3 (VIT) and an Untreated control (UNT). The hybridizations were performed with Affymetrix Human Genome U133 A GeneChips [18]. The dataset was used previously to demonstrate the advantages of applying Principal Component Analysis (PCA) on interaction terms from an Analysis of Variance for a large dataset [19]. The MSC dataset has four dimensions (genes, treatments, time points and replicates). Rather than focussing on the expression values, as in the YCC dataset, we analyze the "Gene-Treatment" two-way interaction matrix from the ANOVA model, described in de Haan et al. [19]. It represents the relations of genes with specific treatments - in this case, we want to extend this to describe the relation of specific GO (sub)classes and treatments. Starting from the interaction matrix, genes are selected having GO annotations, where the corresponding GO classes contain at least 4 genes and have the definition Biological Process. This gives us a data matrix of 4 treatments times 11974 genes. Furthermore, a subset of this set is considered containing 50 relevant GO categories. These categories have been defined beforehand by domain experts.

### Principal Component Analysis and biplots

Principal Component Analysis [8] can be used to give a succinct overview of the structure in a high-dimensional data set using a small number of Principal Components (PCs). These PCs are linear combinations of the original variables X1, X2, ..., Xz, chosen in such a way that PC 1 describes the largest fraction of variation in the data, and subsequent PCs describe maximal portions of the remaining variation. An essential requirement is that all PCs orthogonal to each other. Thus, only the first few PCs need to be considered to get a good overview of the data. In our datasets, the variables X1, X2, ..., Xz represent time points or treatments. The data of *n *objects, each measured at *m *time points or treatments, can be written as an *n *by *m *matrix X. In our case, each object represents the mean profile of a GO category (or subgroup within a GO category identified with preclustering). The matrix X is then decomposed by singular value decomposition (SVD), as follows:(1)

where *U *(an *n *by *n *score matrix) and *V *(an *m *by *m *loading matrix) are orthogonal and Λ is an *n *by *m *matrix containing the so called singular values. The superscript T means the transpose of the matrix *V*. For the biplot [20], the *n *× 2 matrix *U*_2 _of the first 2 PCs represents the objects in the data. The *m *× 2 matrix containing the first 2 loading vectors represents the variables in the data. A biplot is then constructed by plotting *U*_2 _and *V*_2 _in the same graph.

### Combining experimental data with GO information

In order to relate GO information with experimental data, either expression profiles or interaction effects from ANOVA, we should combine both entities. Here, we use matrices to represent expression information (*E*) and GO category information (*G*). In both matrices, rows correspond to genes. Columns in *E *indicate time points (as in the YCC dataset) or interactions (the MSC data). In matrix *G*, columns represent GO terms in a binary coding: if a gene is annotated for a specific GO term the corresponding value in the matrix is 1, otherwise it is 0.

By combining the *E *and *G *matrices, categorical information about groups of genes is obtained:(2)

The resulting data table *X *is then scaled column-wise so that the sum of each column is one. The X matrix can now be considered to contain the mean expression profiles - or interaction profiles - for all GO categories of interest.

Finally, PCA is performed on the X table containing combined information sources. The results can be visualized with a biplot [20]. In the biplots the GO classes are shown as points; cell cycle phases and treatments are indicated as arrows. The biplot allows the GO terms to be correlated with the treatments or the cell cycle phases.

### Preclustering

In order to prevent interesting profiles within one GO category to cancel out, we propose to perform a cluster analysis on all GO categories of interest individually, provided they contain enough genes. The actual clustering is performed on the submatrix of the gene expression matrix E, that corresponds to all columns and genes within a go cluster. Based upon the results of the gene expression clustering, new GO groups (one per cluster) are added to the GO matrix. This step is indicated with the term "preclustering". In the remainder of the paper, we will only consider GO (sub)categories containing at least four genes. In principle, any clustering method can be used. We have chosen to cluster the genes, present in each GO class, with model-based clustering [16] because this is one of the few methods giving an indication of the optimal number of clusters automatically. When this optimal number equals one, the category is not split; if it is larger than one, the GO class is split into several subclasses. Model-based clustering is available in several R packages, such as flexmix and mclust. Which method is used is not very important - the main motivation is to achieve an automatic assessment of the optimal number of clusters. In our scripts, we have used the 2002 version of mclust (available as mclust02). Model-based clustering has been applied to gene expression data before (e.g., [3]). The data are described as a mixture of (normal) distributions. The method applies a number of different models, identified by more or less stringent constraints. Parameters for these models include different variations of shape, volume, and orientation of the clusters. Selection of the best model is performed using the Bayesian Information Criterion (BIC, [21]). This index corrects for several parameters used in the clustering - for instance the number of clusters - to enable a fair comparison between the results of different models. The clustering of the model with the best BIC was chosen with a minimum of 1, and maximum of 8 clusters; it is not to be expected that a higher number of clusters is needed in practice, but one can easily check whether the results improve upon increasing this upper limit. The steps in the preclustering algorithm are displayed below:

1. Construct the E matrix of expression values and the G matrix containing GO category information.

2. Set maximum number of clusters and minimum number of genes in cluster (here 8 and 4 respectively)

3. For each GO class:

3a. select the submatrix from matrix E that contains only the genes from that particular GO class

3b. perform gene-wise clustering of the submatrix and select the best clustering

3c. accept only clusters with at least the minimum number of genes

4. Define a new G matrix containing either the original GO classes or, when clusters have been detected, the GO subclasses - one column is used for each (sub)class

5. Multiply E and the new G matrix to obtain the X matrix

6. Perform column-wise scaling of X by dividing by the number of genes in each subclass

7. Perform PCA on the scaled X matrix and show the biplot

Of course, the best clustering model will not necessarily result in interesting clusters only. From a GO class, clusters which contain noise or have a less interesting profile, can also be formed. Therefore a measure is used to assess the cluster quality of each individual cluster. One way to do this is to calculate the volume for each cluster, available from the model-based clustering. Small volumes indicate tight clusterings. An alternative measure, independent of the clustering method, is to use the mean of all the (Pearson) correlations between the genes within a cluster, the Intra Cluster Correlation (*ICC*):(3)

Here, *C*_*i *_is an element in the lower triangle of the correlation matrix of the genes in a cluster. The measure is also used by Busold et al. [15] but was not specifically given a name. By using the *ICC *it is possible to assess the quality of the newly formed subgroups from a GO class: high values indicate tight clusters. An additional advantage of the *ICC *over a measure like cluster volume is that uninteresting clusters around zero, which may cover a small volume and therefore would seem interesting according to the volume criterion, usually show only low *ICC *values.

The result of the preclustering stage is a new set of GO categories, nested in the original categories. These new categories now are used as matrix *G *in Equation 2 to obtain matrix *X*, the focus of attention. As a consequence, one original GO category now may have several representatives in this matrix, and in that case will show up multiple times in PCA plots. The matrix E containing the expression values is not changed by the preclustering algorithm.

## Results and Discussion

In this section, we will compare the results of PCA using the original GO categories with the results of PCA on the preclustered GO data. Besides the restrictions mentioned previously in paragraph 3.1, all GO classes are taken into account, no cuts were made to exclude parts of the GO tree. In Table 1 the number of GO terms before and after preclustering is summarized for the datasets. Besides the number of unchanged original GO categories, the number of original GO categories split into new subgroups based on the clustering is shown. Furthermore the number of new subgroups containing more than 4 genes is given - these are used for further analysis.

**Table 1:** Number of GO categories, before and after preclustering.

Dataset	# genes	# GO cl.	# split	# new	# > 4	total
YCC	348	68	12	29	14	70
MSC	11,974	922	553	1,933	1,284	1,653
MSC (50 cl.)	1,252	24	24	100	79	79

Only categories containing more than four genes are considered. The header #>4 means the number of groups which contains more than 4 genes.

Clearly, for the MSC data set and the relevant subset of 50 GO categories, many GO categories are heterogeneous with respect to the expression data. For the subset, for example, all GO categories are split, leading to 100 new categories. Of these 100, 79 contain more than four genes. For the much smaller and simpler YCC data set, there is still a group of 12 GO categories that is split, leading in total to 70 categories each containing more than four genes.

### YCC data

**Figure 2:**
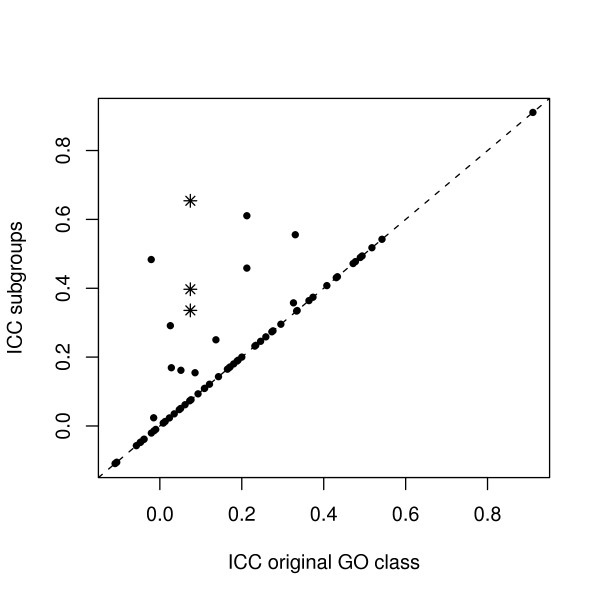
**Plot showing the relation between the *ICC *of the grouped genes from original GO classes (*x*-axis) and the new subgroups (*y*-axis)**. Each point represents a class. The GO term GO:0007042 is marked with three asterisks, one for each subclass.

To investigate whether the new GO subcategories generated with preclustering show relevant cluster structure, the *ICC*s of the original GO classes and the new subgroups are compared in Figure 2. For most classes (56, see Table 1) the *ICC *has not changed. These classes lie on the diagonal, and consist of GO terms for which the optimal number of clusters is determined to be 1: no meaningful subclasses are formed. However, several new subclasses with an increased *ICC *can be observed above the diagonal line. These are examples of groups of genes having a specific profile, which is cancelled out to some extent when the GO category as a whole is taken into account. In some instances, a new subclass with fewer than four genes is found. Such a class will not be taken into account and is not shown in the figure. Note that in this case there is no subclass showing a decrease in *ICC*.

As an example of a heterogeneous GO class benefiting from preclustering the term "cell wall organization and biogenesis" is taken. The three subclasses are shown with asterisks in Figure 2. The expression profiles and clusters for this term can be seen in Figure 1 in the introduction. The term is chosen because it is expected to be involved in the cell cycle, where the cell wall has to be organized and assembled during cell division. The new subclasses generated with clustering have an increased *ICC *compared to the *ICC *of the whole class. The profiles are more specific now and will not cancel out in the analysis.

**Figure 3:**
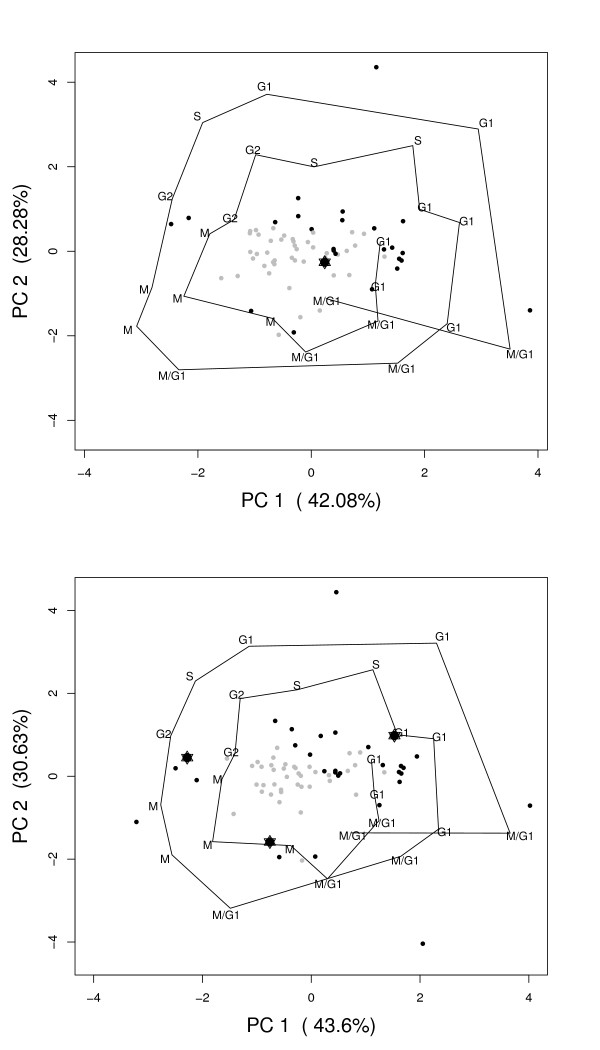
**Visual representation of PCA results for the YCC dataset**. The PCA results without (A) and with preclustering (B) are shown. Several categories for the preclustered PCA are more outward and at a different location than for the PCA without preclustering. Categories (PCA scores) are shown as points, which can be correlated with phases of the cell cycle (connected by lines). Dark points have a *ICC *which is larger than 0.2. Specifically marked is the category "cell wall organization and biogenesis" (GO:0007047, represented by star symbols). Cell phases are indicated by names (G1, S, G2, M and G1/M) also used by [12].

The results of PCA of the YCC dataset with the normal GO classes, and PCA of this dataset after clustering of profiles within GO classes can be seen in Figure 3. This allows for a comparison of the results and shows the advantages of preclustering GO classes in PCA. The 24 time points of the cdc15 experiment are shown as loadings, and are connected by lines. The fact that the cell cycle is passed through more than once results in a large number of time points associated with the same phase (G1, S, G2, M or G1/M). A separation of phases can be seen in both Figure 3A and Figure 3B. The G1 phase is separated from the M and G2 phases on the x-axis and the M phase is separated from the G2 and S phase on the y-axis. The explained variance in the first two Principal Components (PCs) is more than 70 percent.

The consequences of preclustering are shown by a number of differences between Figures 3A and 3B. The term "cell wall organization and biogenesis", represented with a star, is used as an example showing interesting changes. When the whole GO class is used (Figure 3A), the term seems uninteresting and is close to the center of the PCA biplot. It is hard to associate it with a specific phase of the cell cycle. When the different profiles are separated by the clustering however, the new subgroups can be associated with phases G1, M, and between M and G2.

### MSC data

**Figure 4:**
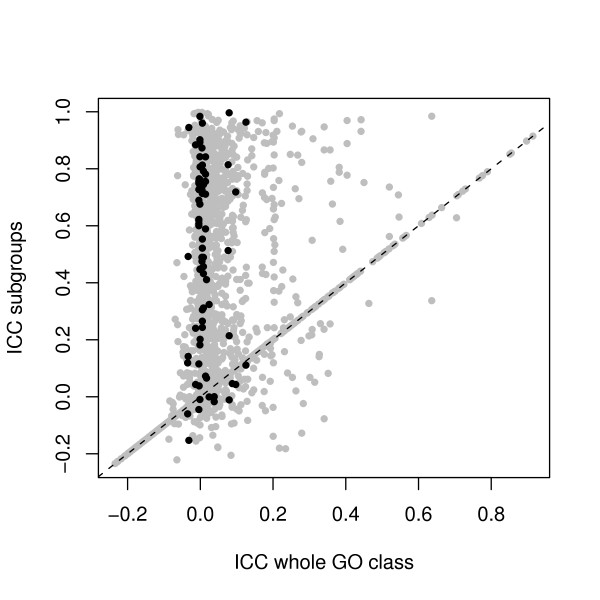
**Representation of the *ICC *for GO categories evaluated with PCA for the MSC dataset**. The *ICC *values of the whole GO class are on the x-axis and the *ICC *of the corresponding subgroup(s) are on the y-axis. A group of classes expected to be involved in the MSC dataset is marked with black dots, other classes are marked with grey dots.

For the MSC dataset similar questions about the comparison between normal PCA and PCA after preclustering of GO classes can be asked. The corresponding *ICC *plot is shown in Figure 4. The original number of 922 GO classes has increased to 1653 subclasses after preclustering, indicated with gray dots. As shown previously, not all classes are divided into subgroups and the unchanged classes are appearing on the diagonal. A considerable number of newly formed classes has an increased *ICC*. Many of these subclasses have a low *ICC *for the original GO classification, which results in vertical band of points at around *x *= 0. The plot also tells us that a number of new subclasses are formed with a lower *ICC *than the original GO classes. These subclasses arise when a group of less related expression profiles remains after relevant profiles are split off in the preclustering.

Because of the large number of GO terms for this dataset we will focus on the terms involved in cell differentiation and osteogenesis. Out of a list of 50 GO terms which could be expected to be responsive to the conditions and the setup of the experiment, 24 terms were present in our dataset 2, taking into account our criteria for the minimum number of genes in a class. For these 24 classes, clustering of the expression profiles in the original GO classes gives rise to 79 classes. These subclasses are marked with black dots in Figure 4. A large number of new subclasses show an increased *ICC*; they all lie in the vertical band described above. There is also a number of new subclasses which has a decreased *ICC*.

**Figure 5:**
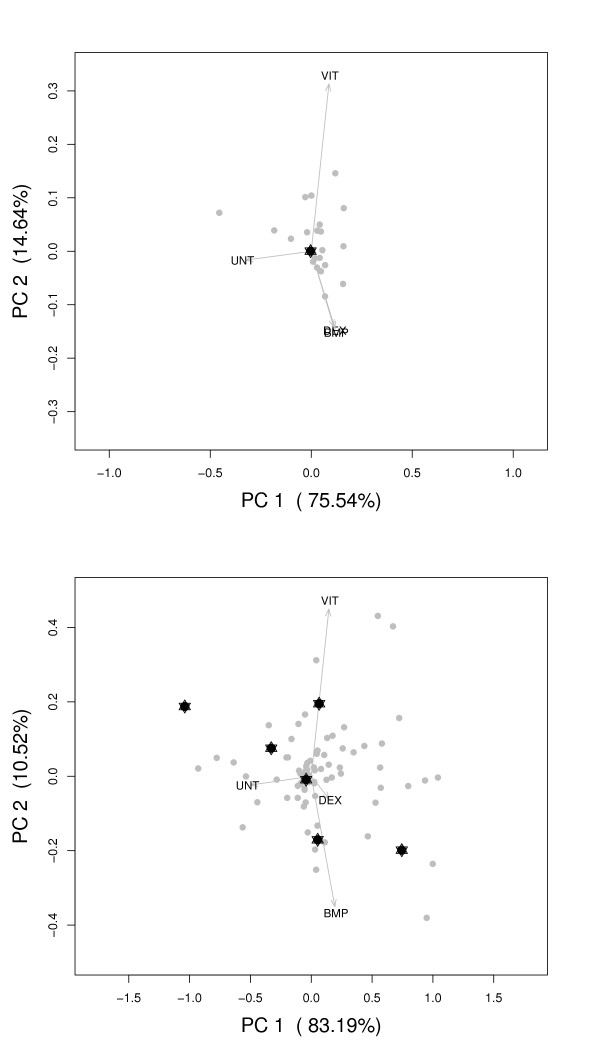
**Visual representation of PCA results for the MSC dataset, without (A) and with preclustering (B)**. The dots (scores) represent GO categories or subgroups, and the arrows (loadings) are the treatments with which the categories can be correlated (indicated with DEX, BMP, VIT and UNT). Only a subset of GO classes is depicted, to focus on cell differentiation and osteogenesis. In Figure 5A the 24 original GO classes are shown and in Figure 5B the 79 subclasses. The stars identify the GO term GO:0001501 (skeletal development).

For the MSC dataset, PCA results with and without preclustering are shown in Figures 5A and 5B. One should remember that in contrast to the YCC dataset, where expression values are used, here, the interaction values from the ANOVA model are analyzed. Four loadings are shown as arrows, corresponding to the three osteogenic treatments and the untreated control (DEX, BMP, VIT and UNT). The first two PCs describe 89.4 percent of the total variation in Figure 5A, and 94.2 percent in Figure 5B. A separation between GO terms associated with the untreated control and the osteogenic treatments can be seen in the first PC for both Figures 5A and 5B. The direction of the UNT arrow is opposite of the three treatments [19]. In the second PC, a separation between VIT and the two other treatments can be observed.

The number of subclasses associated with cell differentiation and osteogenesis has increased from 24 original GO classes to 79 subclasses, as mentioned before. The GO term "skeletal development" is shown as an example of a category which benefits from preclustering. The group is marked with a star symbol in Figure 5A. After preclustering, six subclasses are identified. As can be seen in Figure 5A, the original term could not be associated with a specific treatment and is present in the center of the plot. This has changed dramatically after clustering, as shown in Figure 5B. One subgroup is still in the center of the plot, but the other subclasses can be associated with individual treatments. Two subgroups can specifically be correlated with osteogenic treatments, one for VIT and one for BMP. These groups are even lying on the corresponding arrows for the loadings. A number of genes within the subgroups corresponds with information known from biological literature. The new subgroup lying on the arrow of the BMP contains the gene MSX1 for instance, which has been proven to be induced by BMP in mice [22]. An example of a gene present in the newly formed subgroup lying on the loading of the VIT treatment is MSX2, which is known to be regulated by vitamin D [23]. Finally, there are two subgroups which lie in the direction of the untreated arrow and one final class which could possibly be correlated with DEX.

To give additional statistical evidence of the improvements of preclustering, the identification of the 24 terms mentioned previously was generalized. The generalized procedure was used for both unclustered and preclustered X matrices of the MSC dataset (containing 922 and 1653 profiles, respectively). From the first 2 PCs of the X matrix, interesting profiles were selected from the outside of the scores in steps of decreasing "interestingness". The larger the distance from the center, the more interesting a profile can be. The selection was based on the Mahalanobis distance of a group or subgroup to the center of the data. To assess the relevance of a selection, the set of 24 GO terms in Table 2 is used as a reference. At each step the selectivity and specificity of the selection can be determined from the number of reference GO terms, which are either present or not present in the selection.

**Table 2:** Table of 24 GO terms which were used to generate the ROC plot.

GO term	description
GO:0007050	cell cycle arrest
GO:0009653	morphogenesis
GO:0001558	regulation of cell growth
GO:0045786	negative regulation of progression through cell cycle
GO:0016055	Wnt receptor signaling pathway
GO:0008284	positive regulation of cell proliferation
GO:0016049	cell growth
GO:0009790	embryonic development
GO:0007178	transmembrane receptor protein serine/threonine kinase signaling pathway
GO:0001501	skeletal development
GO:0000188	inactivation of MAPK activity
GO:0030111	regulation of Wnt receptor signaling pathway
GO:0045595	regulation of cell differentiation
GO:0001503	ossification
GO:0000187	activation of MAPK activity
GO:0000165	MAPKKK cascade
GO:0043406	positive regulation of MAPK activity
GO:0030198	extracellular matrix organization and biogenesis
GO:0050793	regulation of development
GO:0007179	transforming growth factor beta receptor signaling pathway
GO:0042127	regulation of cell proliferation
GO:0040007	growth
GO:0051216	cartilage development
GO:0000902	cellular morphogenesis

The terms are present in the genes which were used to generate the mean pro les in the X matrix, before and after preclustering. The terms were selected by biologists and expected to be relevant to the biologic context of the experiment, which is the development of human mesenchymal stem cells to osteoblasts.

**Figure 6:**
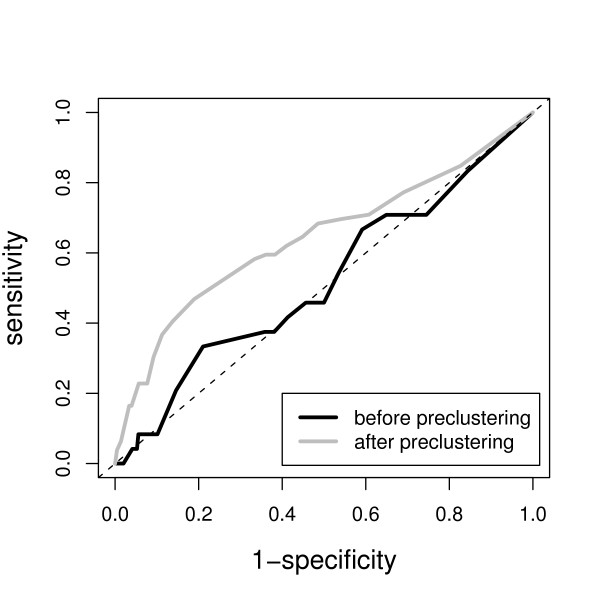
**ROC plot to exemplify the improved identification of interesting terms by performing preclustering**. The sensitivity and specificity of identification of 24 relevant GO terms was calculated to draw the lines. The curve generated from the pleclustered data (grey line) is more sensitive and specific than the original data without preclustering (black line).

Now a comparison between unclustered and preclustered data can be made by drawing ROC curves - see Figure 6. The preclustered method is clearly more sensitive and specific, compared to the data where no preclustering has been performed. This means that newly formed preclustered profiles are more to the outside of the data in the first 2 PCs, and are more readily marked to be interesting.

## Discussion

We have shown a simple and general method to relate expression levels, either directly, or after an ANOVA, to GO categories at all levels in the hierarchy simultaneously. The crucial step is the realization that genes, although they are in the same GO category, may show different profiles. As a result, the application of preclustering leads to more differentiated information. The method is generic; we have opted to use model-based clustering, but in principle any other clustering method can be used, provided that it is possible to automatically generate a reasonable estimate of the number of clusters. The method may be adjusted in a number of ways. The lower limit on the number of genes for a GO category to be considered in the analysis is rather arbitrary. For other problems and datasets this can be adjusted depending on the questions for the specific dataset. The same is true for the cutoff for selecting GO categories. It is possible to inadvertently remove categories which are of interest to your dataset, but are very small.

Other techniques have been proposed for relating measurements to annotation information. An example of relating class information and experimental factors is shown by Jeffery et al. [24], who use transcription factor binding sites information. These are sites in the promoters of genes to which a transcription factor can bind. The transcription factors play an important role in the regulatory networks of gene expression. A method called Correspondence Analysis (CA; [25]) was applied to relate classes and experimental information. CA is similar to PCA; the algorithm can reduce the multidimensional data matrix to a lower dimension containing the important information from the data. In the standard way of applying CA, the dependency of values in the rows on the columns of a contingency table for two sets of conditions is investigated. After scaling of both the rows and columns, PCA is applied to the scaled data.

For the analysis of gene expression data, the first articles which applied CA treated the matrix with expression values as a contingency table. The aim was then to associate genes with variables. Kishino et al. [26] applied CA to show the relationship between genes and tissues of a dataset which was designed to investigate colon cancer [27]. Fellenberg et al. [28] also showed the possibility to investigate the associations between genes and variables. The application of CA to incorporate GO information as class information in the visualization of results was shown by Busold et al. [15]. Datasets investigating glucose metabolism and from human pancreatic adenocarcinomas were used.

For our data, CA and PCA would lead to very similar results, because of the fact that the column means and row means, used for scaling in CA, are very similar, something that is also true for the data used in, e.g., [28]. In Figure 6, the CA curves would completely overlap with the PCA curves and therefore have been left out. Since PCA is more easy to interpret, we prefer it for this case. Preclustering of GO information as presented here could also reveal interesting findings otherwise discarded in methods using class information like CA.

GO information (and other annotation information) is to some extent limited, because not all genes are annotated. This will have a limiting effect on the size of clusters for less well known GO categories; if a certain GO category in the database is not described correctly, or extensively enough, it will obviously be hard to link this GO category with the experimental factors of interest. However, the number of annotations will increase rapidly so that the method will only gain in importance.

With the division of GO classes in clusters with similar profiles, it is possible that one of the new classes gets a high *ICC*, but remains in the center of the biplot. Such a subgroup will contain relatively flat profiles; nevertheless, it is beneficial that it is clustered separately, to prevent other interesting subgroups to be obscured. One final result of the preclustering is that a category can be found more than once in the same PCA biplot. This can make it more difficult to draw conclusions for a single GO category as a whole. On the other hand, the results will be more indicative of what is really happening within a GO class.

## Conclusions

The advantages of clustering heterogeneous GO classes have been shown here for two real gene expression datasets, the well-known YCC dataset and the MSC dataset. The results show that preclustering yields an increased number of interesting groups deviating from the center of the PCA biplot. In this center the less interesting groups with flat profiles are present. In future, more formal selection criteria could be used to identify interesting GO classes and newly formed subclasses, based on statistical significance.

These properties of preclustering allow for a better association of GO categories with phases or treatments, because interesting subgroups which are obscured by different profiles are separated from each other. New meaningful relations are discovered which would not have been found otherwise with PCA. For the GO processes "cell wall organization and biogenesis" (GO:0007047) and "skeletal development" (GO:0001501) this is explicitly shown.

## Authors' contributions

JdH participated in the design of the study, conceived and performed the statistical analyses, and drafted the manuscript. EP, RCvS, and JCdV helped in the biological analysis of the results. SB participated in generating the MSC data set and in the design of the study, and helped in the biological analysis of the results. LMCB participated in the design of the study and helped in the statistical analysis of the results. RW participated in the design of the study, helped in the statistical analysis of the results, and drafted the manuscript. All authors read and approved the final manuscript.
